# Exploring the influence of deposit mineral composition on biofilm communities in oil and gas systems

**DOI:** 10.3389/fmicb.2024.1438806

**Published:** 2024-07-30

**Authors:** Maria A. Diaz-Mateus, Silvia J. Salgar-Chaparro, Johanna Tarazona, Hanan Farhat

**Affiliations:** ^1^WA School of Mines: Minerals, Energy and Chemical Engineering, Curtin Corrosion Centre, Curtin University, Bentley, WA, Australia; ^2^Qatar Environment and Energy Research Institute (QEERI), Doha, Qatar

**Keywords:** mineral deposits, oil and gas, microbial community structure, biofilm, extracellular polymeric substances

## Abstract

**Introduction:**

Inside oil and gas pipelines, native microbial communities and different solid compounds typically coexist and form mixed deposits. However, interactions between these deposits (primarily consisting of mineral phases) and microorganisms in oil and gas systems remain poorly understood. Here, we investigated the influence of magnetite (Fe_3_O_4_), troilite (FeS), and silica (SiO_2_) on the microbial diversity, cell viability, biofilm formation, and EPS composition of an oil-recovered multispecies consortium.

**Methods:**

An oilfield-recovered microbial consortium was grown for 2 weeks in separate bioreactors, each containing 10 g of commercially available magnetite (Fe_3_O_4_), troilite (FeS), or silica (SiO_2_) at 40°C ± 1°C under a gas atmosphere of 20% CO_2_/80% N_2._

**Results:**

The microbial population formed in troilite significantly differed from those in silica and magnetite, which exhibited significant similarities. The dominant taxa in troilite was the *Dethiosulfovibrio* genus, whereas *Sulfurospirillum* dominated in magnetite and silica. Nevertheless, biofilm formation was lowest on troilite and highest on silica, correlating with the observed cell viability.

**Discussion:**

The dissolution of troilite followed by the liberation of HS^−^ (H_2_S) and Fe^2+^ into the test solution, along with its larger particle size compared to silica, likely contributed to the observed results. Confocal laser scanning microscopy revealed that the EPS of the biofilm formed in silica was dominated by eDNA, while those in troilite and magnetite primarily contained polysaccharides. Although the mechanisms of this phenomenon could not be determined, these findings are anticipated to be particularly valuable for enhancing MIC mitigation strategies currently used in oil and gas systems.

## 1 Introduction

In natural environments, microorganisms tend to attach to surfaces since it supposes an ecological advantage over the planktonic lifestyle (Zobell, 1943). Biofilms enable microorganisms to thrive in hostile environments; their 3D structure allows nutrient circulation and encourages symbiotic relationships and the exchange of genetic material between cells (Costerton et al., 1999). An established biofilm structure consists of microbial cells and extracellular polymeric substances (EPS), natural polymers secreted by microorganisms during growth that offer structural stability to the microorganism attached to a surface (Karygianni et al., 2020). EPS, which primarily consists of macromolecules such as proteins, polysaccharides, nucleic acids, and lipids, are known to act as a physical barrier that can concentrate diverse substances at the biofilm-surface interphase, and facilitate the creation of microenvironments that are radically different from the bulk aqueous phase in terms of pH, dissolved oxygen, organic and inorganic species (Little et al., 2015). EPS can also impact the diffusion of various molecules from the aqueous phase into the biofilm, creating nutritional gradients across the biofilm (Karygianni et al., 2020). Biofilm formation harms different sectors, such as medical health, food and agriculture, water and wastewater, oral care, and energy (Cámara et al., 2022). In the energy sector, biofilms are responsible for a type of corrosion termed microbiologically influenced corrosion (MIC) that occurs in metallic assets as a consequence of the creation of aggressive microenvironments at the metal-biofilm interphase that cause localized damage (Videla and Herrera, 2005).

Recently, in oil and gas systems, a relatively new MIC mechanism termed under deposit microbial corrosion (UDMC) has been observed. UDMC refers to the corrosion caused by the combined presence of deposits and microorganisms and has been associated with pipeline failures in different case studies (Esan et al., 2001; Bruijnen et al., 2020). Different solid compounds can be present in production wells, depending on the water’s chemistry conditions, temperature, and pressure. Some of these solid compounds, or deposits, are formed as a precipitation product when the concentrations of ions that form soluble salts exceed their saturation conditions, others are materials formed as a result of metal oxidation (corrosion), and others can enter the system directly from the formation reservoir (Little et al., 2015; Brown and Moloney, 2017). Although the different authors that have studied UDMC in laboratory-based studies suggest that deposits facilitate the colonization and protection of microorganisms on metal surfaces, thereby increasing the risk of MIC through the accumulation of corrosive metabolites beneath the deposit, to the best of the author’s knowledge, to date, there is no supporting data for this claim. Properly validating this hypothesis requires separate studies on the interactions of microorganisms with the deposits found in oil and gas systems and the corrosion mechanisms by the deposits alone and microorganisms alone.

Most deposits found in oil and gas systems are minerals with a chemical formula and an ordered atomic structure. For instance, in sour environments, i.e., environments with high concentrations of H_2_S, the most commonly found deposits are the iron sulfides mackinawite, pyrite, troilite, and pyrrhotite (Smith, 2015). Conversely, in sweet environments, i.e., environments with high concentrations of CO_2_, a wide range of different deposits have been reported; calcite, siderite, magnetite, and chukanovite are some of them (Pandarinathan et al., 2014; Hua et al., 2019; Li W. et al., 2023; Li X. et al., 2023; Owen et al., 2023). Moreover, most microbial species recovered from oil and gas systems have also been isolated from natural mineral-rich environments, such as hydrothermal vents, cave systems, sedimentary environments, mine environments, and soil (Teske et al., 2000; Parker C. W. et al., 2018; Parker S. P. et al., 2018; Martinez-Alonso et al., 2019; Allenby et al., 2022). And despite metal-microbe interaction being a matter widely studied in the area of bioleaching, there are still major knowledge gaps within the field of biogeochemistry that require further research, such as the behavior of multispecies consortium, the integration of mineral and microbial ecology, and the definition of mineral biosignatures (Dong et al., 2022). More importantly, in the specific area of MIC, the interaction of microorganisms with mineral deposits is a poorly explored matter.

Our study aimed to determine to which extent the presence of a specific mineral phase in a simulated oil and gas system drives the composition of microbial communities. Furthermore, we aimed to understand the influence of deposits mineral composition on the viability of sessile cells, formation of biofilms and EPS composition. Investigating the interactions of microorganisms and different types of mineral deposits in oil and gas production facilities is crucial for developing more effective mitigation strategies against MIC and establishing reliable MIC risk assessments.

## 2 Materials and methods

### 2.1 Microbial recovery and consortium preparation

The microbial consortium used in this study was sourced from sand deposits within a high-pressure separator at an Australian oil production facility. Five grams of sand were inoculated in 100 mL vials containing various selective culture media to optimize the recovery of diverse microbial groups associated with corrosion processes. The culture media targeted the growth of sulfate-reducing bacteria (SRB), thiosulfate-reducing bacteria (TRB), methanogenic archaea (MET), acid-producing bacteria (APB), iron-reducing bacteria (IRB), iron-oxidizing bacteria (IOB), and general heterotrophic bacteria (GHB). Following the guidelines outlined in the NACE standard TM0194 (NACE International, 2014) and the composition described by Salgar-Chaparro et al. (2020a,b), for culture media supporting sulfide-producing prokaryotes (SPP), the media were prepared, autoclaved (121°C, 20 min) and deaerated with a sterile gas mixture of 20% CO_2_ and 80% N_2_. Incubation occurred at 40°C to replicate field conditions. To establish the microbial consortium for our study, the culture media demonstrating growth after 10 days of incubation were combined in equal proportions. The microbial community profiling determined by 16S rRNA gene sequencing revealed that the inoculum was predominantly composed of members of the genus *Pseudomonas*, unclassified *Enterobacteriaceae*, *Ercella*, and unclassified *Petrotogacaeae* (Supplementary Figure S1).

### 2.2 Evaluation of the impact of deposits on biofilm characteristics

#### 2.2.1 Mineral deposits

To replicate the key components found in deposits commonly encountered in oil and gas pipelines, we obtained three inorganic minerals—iron oxide, silicon dioxide, and iron sulfide—from Sigma-Aldrich (St. Louis, United States) with analytical reagent-grade quality. These minerals served as the representative deposits in our study. To ensure these representative deposits’ sterility, iron oxide, and silicon dioxide underwent autoclaving at 134°C for 3 min at 208 kPa before the experimental setup. In contrast, iron sulfide was sterilized through gamma-ray radiation (50 kGy) over 56 h. Because of the acknowledged susceptibility of iron sulfide to oxidation under elevated temperatures, pressure, and humidity, gamma irradiation was selected as the appropriate sterilization technique (Menendez et al., 2013; Robineau et al., 2021; Li W. et al., 2023; Li X. et al., 2023).

The specific surface area of the minerals was determined using the Brunauer, Emmett, and Teller (BET) method (determined by multipoint BET N_2_ adsorption) (Brunauer et al., 1938). In addition, the particle size distribution of the three minerals was done by laser diffraction analysis; results are presented in Table 1. Likewise, the mineral phases of the iron oxide, silicon dioxide, and iron sulfide were examined via powder X-ray diffraction (XRD) and identified as magnetite (Fe_3_O_4_), silica (SiO_2_), and troilite (FeS), respectively (Supplementary Figure S2).

**Table 1:** Mean particle size and specific surface area of the deposits investigated.

Model deposit	Mean particle size (μm)	Specific surface area (m^2^/g)
Iron oxide (Fe_3_O_4_)	4.67	5.41
Silicon dioxide (SiO_2_)	4.55	6.48
Iron sulfide (FeS)	62.29	0.68

#### 2.2.2 Incubation experiment

The incubation experiments for the three different minerals were performed in 2-liter capacity glass cells. For each deposit, 10 g were evenly distributed among five custom-made glass containers (Φ20 × 10 mm) inside each reactor, resulting in 2 g per sample container and five replicates for microbiological analysis. A synthetic produced water solution was used for the incubation experiment. Briefly, 1 L of the test solution consists of: 20 g/L of sea salts (Millipore), 20 mM of Na-lactate, 20 mM Na-acetate, 20 mM Na-formate, 7.1 mM glucose, 1.1 g/L bacto casamino acids (BD), 20 mM NH_4_NO_3_, 10 mM Na-thiosulfate, 1 mM FeCl_2_·4H_2_O, 1 mM MnCl_2_·4H_2_O, 1 L of ultrapure deionized water. The solution was buffered with sodium bicarbonate, and the initial pH was 7.3 ± 0.2, mirroring field conditions.

To maintain anaerobic conditions throughout the exposure period, the reactors were continuously sparged with a filtered-sterilized gas mixture of 20% CO_2_/80% N_2_ at a rate of 20 mL/min. The microbial consortium was added to the reactors at a final concentration of 10^7^cell/mL. The temperature and stirring conditions were set to 40°C ± 1°C and 200 rpm, respectively. Continuous feeding was implemented to ensure the vitality of the microbial consortium during the two-week exposure period, with 30% of the test solution volume replenished every 24 h.

#### 2.2.3 Monitoring of dissolved iron and sulfides in test solution

Microbial metabolism (reduction) of thiosulfate in the test solution was measured every 3 days using spectrophotometry (Hach^™^, DR3900). Total dissolved iron (FeT) concentration was determined by the USEPA FerroVer^®^ method following the manufacturer’s instructions. Likewise, sulfide levels were assessed using the Hach^™^ method 8131 (Methylene Blue Method) following the manufacturer’s instructions.

#### 2.2.4 Microbial community composition

Triplicate samples (1 g) from each deposit underwent simultaneous DNA and RNA extractions at the experiment’s conclusion using the Norgen DNA/RNA/Protein kit (Norgen Biotek Corp), following the manufacturer’s protocols. After RNA extraction, genomic DNA was eliminated from the samples using the Turbo DNA-free kit (Invitrogen) per the manufacturer’s instructions. RNA was then purified using an RNeasy MinElute cleanup kit (Qiagen) and transcribed into complementary DNA (cDNA) using the SuperScript IV first-strand synthesis system (Invitrogen), as previously described (Salgar-Chaparro et al., 2020a,b).

The eluted DNA and synthetised cDNA were sent to the Marshall Centre at the University of Western Australia for 16S rRNA gene amplicon sequencing of the V3-V4 hypervariable region (Yu et al., 2005). Sequencing was conducted on a MiSeq sequencing instrument (Illumina).

Raw FASTQ files were joined, demultiplexed, and classified into amplicon sequence variants (ASVs) using the “dada2 denoise-paired” plugin on the Quantitative Insights Into Microbial Ecology Software (QIIME 2) (Callahan et al., 2016; Bolyen et al., 2019). The DADA2 quality settings “- -p-trunc-len-f 280” and “- -p-trunc-len-r 220” were applied to truncate the forward and reverse sequences at 280 and 220 positions, respectively. We referenced the Silva138 database for taxonomy assignment and taxonomy table generation (Bokulich et al., 2018). Sequencing data was analyzed using R (v4.2.1) and RStudio (v2022.02.3). The relative abundance of specific microbial groups in the total and active communities was studied at the genus level using the “phyloseq” R package. Bart charts of the microbial communities with phylogenetic groups with relative abundances equal to or greater than 1% were created using the “ggplot2” R package (Wickham and Wickham, 2016).

#### 2.2.5 Microbial diversity

Amplicon Sequence Variants (ASVs) were rarefied to a depth of 10,194 reads, corresponding to the minimum read count among samples in the dataset. Alpha and beta diversity measurements were determined using the phyloseq and microbiome R packages. Alpha diversity was calculated in terms of species richness, employing the Chao1 index, and diversity, using the Shannon index. Beta diversity was estimated based on the Weighted UniFrac distance (Lozupone and Knight, 2005), and a non-metric multidimensional scaling (NMDS) was performed to visualize the multivariate dispersion of the community composition under each condition. A permutational analysis of variance (PERMANOVA) was conducted to test for significant differences in beta diversity. The results of statistical tests were considered significant with *p* ≤ 0.05.

#### 2.2.6 Microbial viability

The culture-dependent method most probable number (MPN) was used to determine the number of viable sessile microorganisms developed on each deposit at the end of the immersion period. Three 1-gram replicates of each deposit were placed in three separate falcon tubes containing 10 mL of phosphate-buffered saline solution (PBS) with Tween 20 (0.1% w/v final concentration). Sessile bacteria were detached from the deposits by vortexing at full speed for 10 s and sonication for 2 min in cycles of 15 s on and 10 s off. An aliquot of 1 mL of the PBS solution (from each falcon tube) containing the sessile microorganisms was inoculated into 9 mL of the fresh test solution and serially diluted 10-fold in triplicate for the MPN estimation. The serial dilutions were incubated at 40°C for 20 days, and microbial growth was determined to be positive based on visually noticeable changes in the turbidity and color of the culture media. When necessary, confirmation by light microscopy for microbial morphologies was performed. The microbial concentration was calculated using the MPN 3-tube standard table (Da Silva et al., 2018). Sessile bacteria counts were expressed as cells per gram of deposit (cell/g). The remaining 9 mL of PBS solution containing detached cells was used to estimate microbial activity levels.

#### 2.2.7 Microbial activity levels and stress measurements

The microbial activity of the sessile microbial communities developed on each deposit at the end of the immersion period was determined in triplicate by measuring the intracellular concentrations of adenosine triphosphate (ATP), adenosine diphosphate (ADP), and adenosine monophosphate (AMP). The AXP assay and the Quench-Gone Organic Modified (QGO–M) test kits (LuminUltra^™^) were employed for this purpose, according to the manufacturer’s instructions. The concentrations of these adenosine nucleotides in the PBS solution containing the detached sessile microorganisms were determined by luminescence using a luminometer (LuminUltra^™^, PhotonMaster) after reaction with the luciferin-luciferase enzyme.

The Adenylate energy charge (AEC), as a measurement of the stress levels of the sessile microorganisms, was calculated according to the AXP test kit (LuminUltraTM) formula:

$AEC=\left(ATP+0.5ADP\right)/\left(ATP+ADP+AMP\right)\text{.}$

#### 2.2.8 Biofilm characterization

##### 2.2.8.1 Confocal laser scanning microscopy

Biofilm extracellular polymeric substances (EPS) of the biofilms formed on the three deposits were characterized using Confocal Laser Scanning Microscopy (CLSM). Triplicate mineral samples were extracted from each reactor and placed in sterile falcon tubes for subsequent staining.

Fluorescent staining was performed to visualize different biofilm components. The first stain used was Sypro^®^ Orange (Thermo Fisher) in a 5X concentration to visualize proteins. The second stain was DiYO^™^-1 (AAT Bioquest Inc.), used according to the manufacturer’s instructions to visualize extracellular DNA (eDNA). Finally, total polysaccharides were visualized using Wheat Germ Agglutinin (WGA) (Thermo Fisher, WGA-Alexa Fluor^™^ 633 conjugate) and Concanavalin A (ConA) (Thermo Fisher, ConA-Alexa Fluor^™^ 633 conjugates) at 50 μg/mL and 100 μg/mL working concentrations, respectively. Stains were combined in ultrapure deionized waterwater and applied to coupon surfaces for 20 min before rinsing with PBS to remove unbound stains, and then transferred to a μ-Slide 2 Well Glass Bottom microscope slide (ibidi, Gräfelfing, Germany).

CLSM imaging was performed on a Nikon A1+ confocal microscope in Z-stack mode, with 3.5–7 micron intervals, using both ×10 and ×20 objectives. Z-stack CLSM images were further analyzed using IMARIS v 9.7 (Oxford Instruments, Oxford, UK) to enhance visualization of biofilm proteins, eDNA, and polysaccharides.

##### 2.2.8.2 Spectrophotometry

To complement the microscopic analysis of biofilms, a Crystal Violet assay was conducted in duplicate to evaluate the biofilm density formed in the presence of three different deposits. The methodology followed the procedure outlined by O’Toole (O'Toole, 2011). One gram of mineral from each reactor was evenly distributed among eight wells of 24-well microtiter plates. As a control, 0.5 g of sterile mineral were distributed equally in four wells. Crystal violet solution at a concentration of 0.5% in ethanol was added to each well in a 100 μL volume and allowed to stain for 20 min. Afterward, wells were carefully rinsed with sterile PBS thrice to remove unbound crystal violet. To release the bound crystal violet, 33% acetic acid was added, and the optical density of biofilms and control samples was measured at a wavelength of 595 nm. This assay provided a quantitative measure of biofilm density, complementing the qualitative insights from the microscopic analysis.

### 2.3 Statistical analysis

To evaluate the intracellular adenylate concentrations (ATP, ADP, and AMP) and alpha diversity across the different experimental scenarios, a comprehensive statistical analysis was conducted utilizing PAST (Version 4.10). The normality of each variable within the dataset was assessed using the Shapiro–Wilk method. A one-way analysis of variance (ANOVA) was performed in conjunction with Tukey’s *post-hoc* means separation test to determine whether statistically significant distinctions existed among normally distributed variables. The results of statistical tests were considered significant when the *p*-value was either equal to or below 0.05.

## 3 Results

### 3.1 Dissolved iron and sulfides monitoring

**Figure 1:**
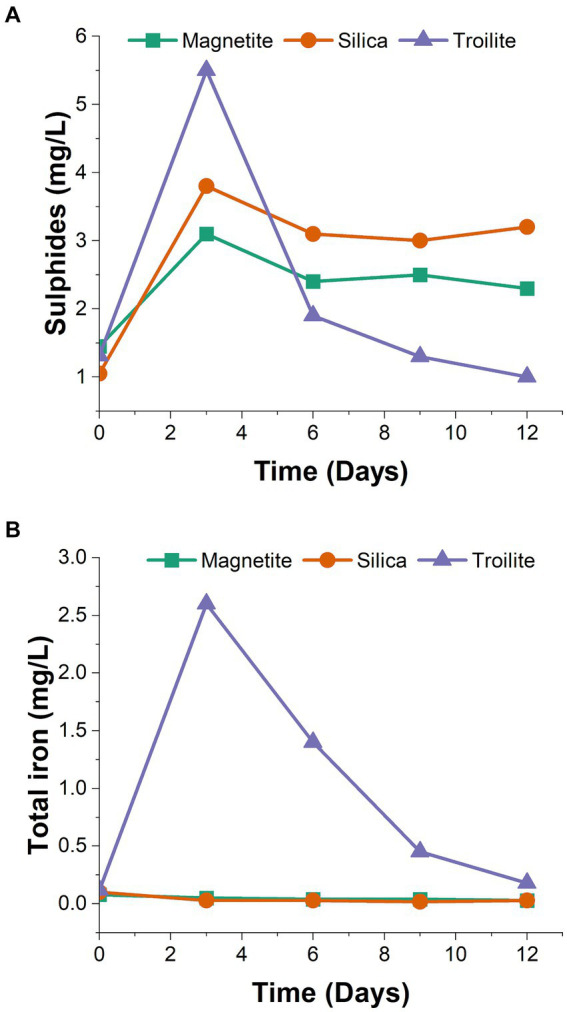
Test solution chemical monitoring at different time intervals. **(A)** Dissolved sulfides (H_2_S, HS^−^) determined by spectrophotometry using the methylene blue method. **(B)** Total iron determined by spectrophotometry using the USEPA FerroVer® method.

The trends in dissolved sulfides and total iron concentrations in the bulk solution of the three rectors over the immersion period are shown in Figure 1. Notably, the reactor containing troilite exhibited the highest concentrations among the three tests for dissolved sulfides and total iron on day 3. This observation and a noticeable color change in the test solution from day 2 to day 3 suggest a chemical reaction occurred in this reactor.

The concentrations of dissolved sulfides (Figure 1A) revealed that the reactor with silica consistently had higher sulfide concentrations than the reactor with magnetite throughout the two-week immersion period. Despite the reactor with troilite showing the highest sulfide concentration in the initial sampling, subsequent samplings revealed it had the lowest sulfide concentrations for the rest of the test.

Regarding total dissolved iron concentrations (Figure 1B), the reactors with silica and magnetite maintained concentrations within the same range as the solution blank (0.03–0.05 mg/L). Contrastingly, the total iron concentrations measured throughout the immersion period in the reactor with troilite indicated iron leaching into the test solution. The decrease in measured iron concentration in the reactor with troilite over time suggests mineral oxidation likely occurred between days 2 and 3, coinciding with the observed color change. Subsequently, the continuous flow of the test solution appeared to wash away the excess iron in the solution.

### 3.2 Effect of deposit type on the microbial community characteristics

#### 3.2.1 Microbial taxonomic profile

The total and active microbial community within biofilms grown in magnetite, silica, and troilite were identified through 16S DNA and RNA-based amplicon sequencing. While DNA sequencing reveals the relative abundance of all microorganisms, including active, dormant, and, in some cases, dead cells, RNA sequencing discloses the relative abundance of only active cells (Salgar-Chaparro and Machuca, 2019).

**Figure 2:**
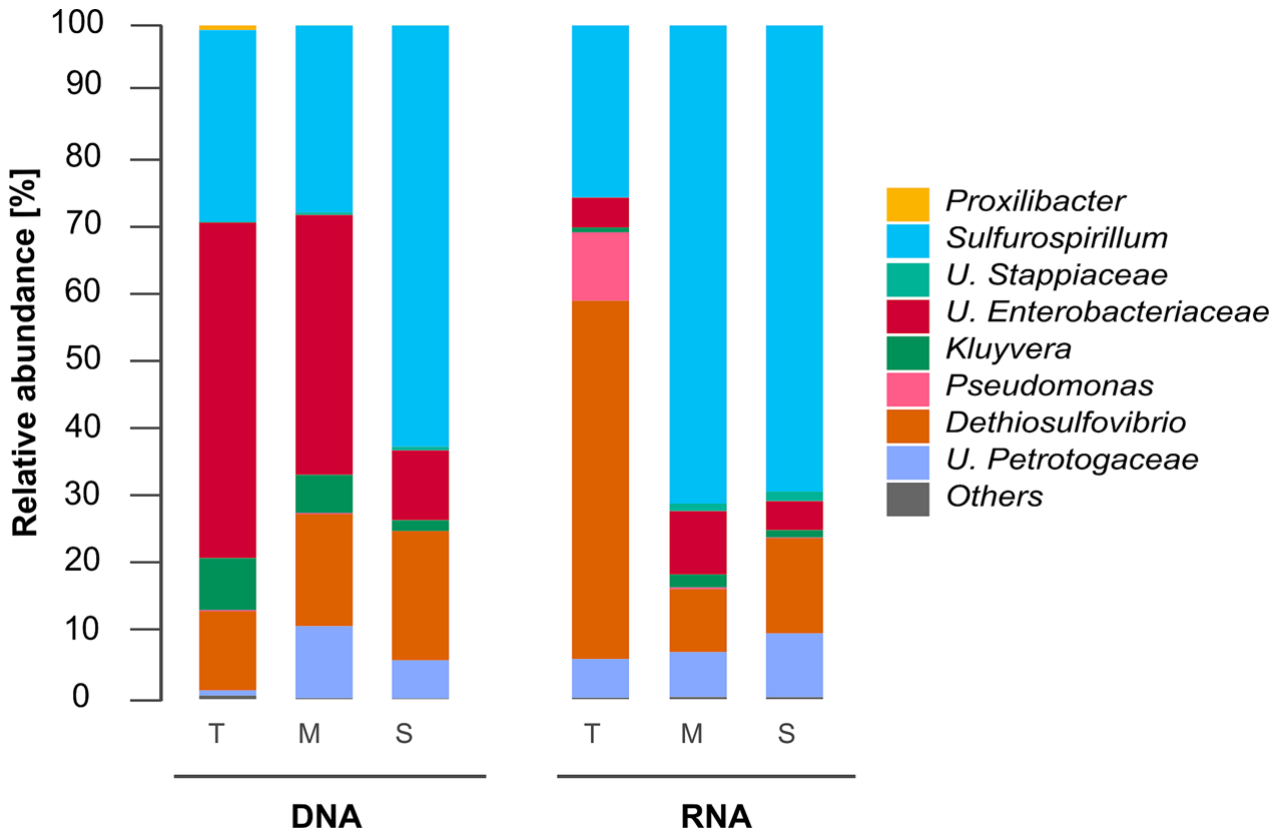
Total (DNA-based) and active (RNA-based) microbial community composition of biofilms grown in troilite (T), magnetite (M), and silica (S). Mean relative abundances of microbial populations classified at the genus level from 16S rRNA sequencing (*n* = 3). Genera with relative abundances lower than 1% in all samples were grouped in the “Others” category.

A total of 2,463,789 reads were generated during the sequencing process, averaging 28,273 reads per sample replicate, with each sample processed in triplicate. Quality filtering of the sequencing reads resulted in 1,323,689 reads for further analysis. These sequences were taxonomically classified into eight microbial genera. The relative abundance of microbial populations at the genus level as a function of the deposit type is shown in Figure 2. Only genera with relative abundances ≥1% are presented. Microorganisms with a relative abundance of less than 1% in the microbial community were grouped as “Others <1%.” The relative abundance of microbial populations and the phylum and class levels as a function of the deposit type are shown in Supplementary Figure S3.

Figure 2 shows that the microbial community composition of biofilms grown in the three different deposits shares the same microbial populations, except for *Proxilibacter* in the reactor with troilite, which was not detected in either the reactors with magnetite and silica. The total communities in the reactors with troilite and magnetite were dominated by *Unclassified Enterobacteriaceae* (57 and 44%, respectively). In contrast, the reactor with silica had a lower relative abundance of this genus (11%). The relative abundance of *Sulfurospirillum* showed an opposite pattern, with lower abundances in the reactors with troilite and magnetite (28 and 27%, respectively) and a higher abundance in the reactor with silica (62%). The *Dethiosulfovibrio* genus exhibited similar relative abundances in the three total microbial populations, accounting for 12, 16, and 19% in the reactors with troilite, magnetite, and silica, respectively. The *Kluyvera* genus showed higher abundances in the reactors with troilite and magnetite (8 and 6%, respectively) and lower abundance in the reactor with silica (2%). Finally, the *Unclassified Petrotogaceae* was more abundant in the reactor with magnetite (11%) than in the reactors with silica and troilite (5 and 1%, respectively).

Similarly, differences in the relative abundance of dominating genera in the active microbial communities were noted. The active community in the reactor with troilite was dominated by *Dethiosulfovibrio* (53%), while in the reactors with magnetite and silica, this genus had lower relative abundances (9 and 14%, respectively). The active communities in the reactors with magnetite and silica were dominated by the *Sulfurospirillum* genus, with 71 and 70%, respectively, whereas this genus had a relative abundance of 25% in the reactor with troilite. Notably, the *Pseudomonas* genus showed a 10% relative abundance in the reactor with troilite, only 0.25% in the reactor with magnetite, and less than 1% in the reactor with silica, included in the “Others” category. The *Unclassified Petrotogaceae* had similar relative abundances in the three active microbial populations, accounting for 6, 7, and 9% in the reactors with troilite, magnetite, and silica, respectively.

The comparison of the DNA and RNA profiles showed marked differences in the relative abundances of total and active populations in the reactors with troilite and magnetite. The total community in the reactor with troilite was dominated by *Unclassified Enterobacteriaceae,* whereas, in the active community, this genus accounted for only 8%, with the *Dethiosulfovibrio* genus being the most abundant. The *Proxilibacter* genus was present in the total microbial community in the reactor with troilite (0.6%) but absent in the active microbial community. Moreover, the relative abundance of the *Pseudomonas* and *Unclassified Petrotogaceae* genera in the total community was only 0.18 and 0.7%, whereas in the active community, they accounted for 10 and 6%, respectively. The total community in the reactor with magnetite was dominated by *Unclassified Enterobacteriaceae* (57%), whereas, in the active community, this genus accounted for only 17%, with the *Sulfurospirillum* genus being the most abundant (71%). Additionally, the relative abundance of *Unclassified Stappiaceae* in the total community in the reactor with magnetite was only 0.3%, whereas in the active microbial community, it accounted for 1.1%.

#### 3.2.2 Microbial diversity

**Figure 3:**
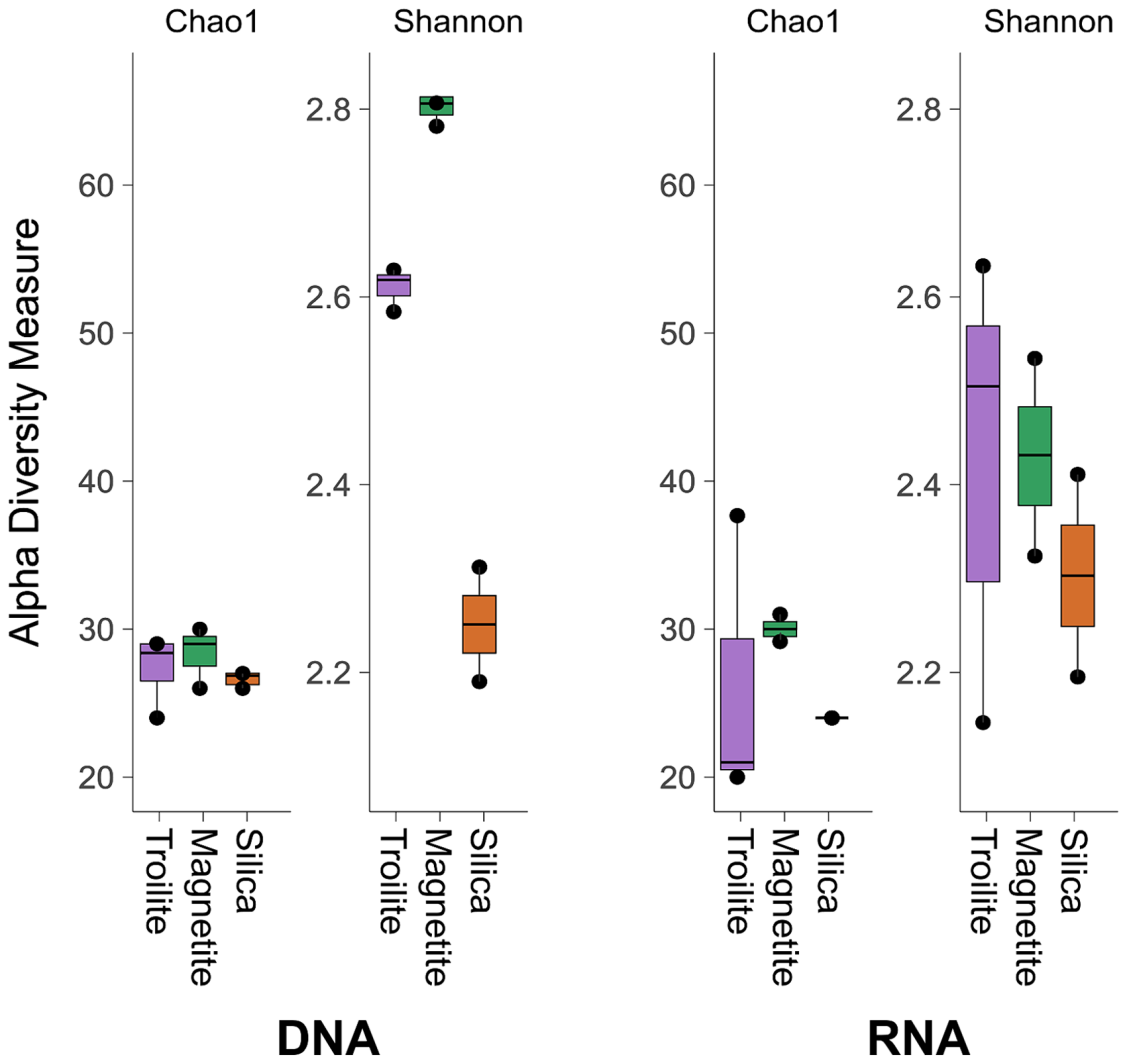
Alpha diversity indices Chao1 and Shannon of total and active microbial communities in the three evaluated deposits.

The impact of different deposits on the alpha diversity of the total and active microbial communities was evaluated using the richness index Chao1 and the diversity index Shannon, as illustrated in Figure 3. The richness index (Chao1) of the microbial community developed in each of the three reactors, reflecting the number of taxonomic groups, was similar for both DNA and RNA-based profiling. This result is in accordance with Figure 2, which indicates that the microbial community compositions in biofilms from the three deposits share the same number of microbial genera. On the other hand, the diversity index (Shannon), which refers to the number of taxonomic groups and their distribution of abundances, was higher in the DNA-based profiling compared to the RNA-based profiling, given the more homogeneous distribution of the relative abundance of different genera observed in Figure 2. Notably, the diversity in the reactor with silica was consistent between DNA and RNA-based profiling.

The Kruskal-Wallis statistical analysis of alpha diversity measurements demonstrated significant variations in richness (Chao1 index) among the three deposits within the active microbial community. Specifically, biofilms grown on magnetite exhibited the highest richness, while the ones grown on troilite showed the lowest; this difference is likely related to the microbial genus present in a relative abundance of <1% (Others). In contrast, no such differences were observed in the total microbial community (*p* < 0.05, Supplementary Table S1). Conversely, statistical differences in diversity (Shannon index) among the three deposits were evident in the total microbial community but not in the active microbial community (*p* < 0.05, Supplementary Table S1). The biofilm grown in magnetite displayed the highest diversity, while the one grown in silica exhibited the lowest.

**Figure 4:**
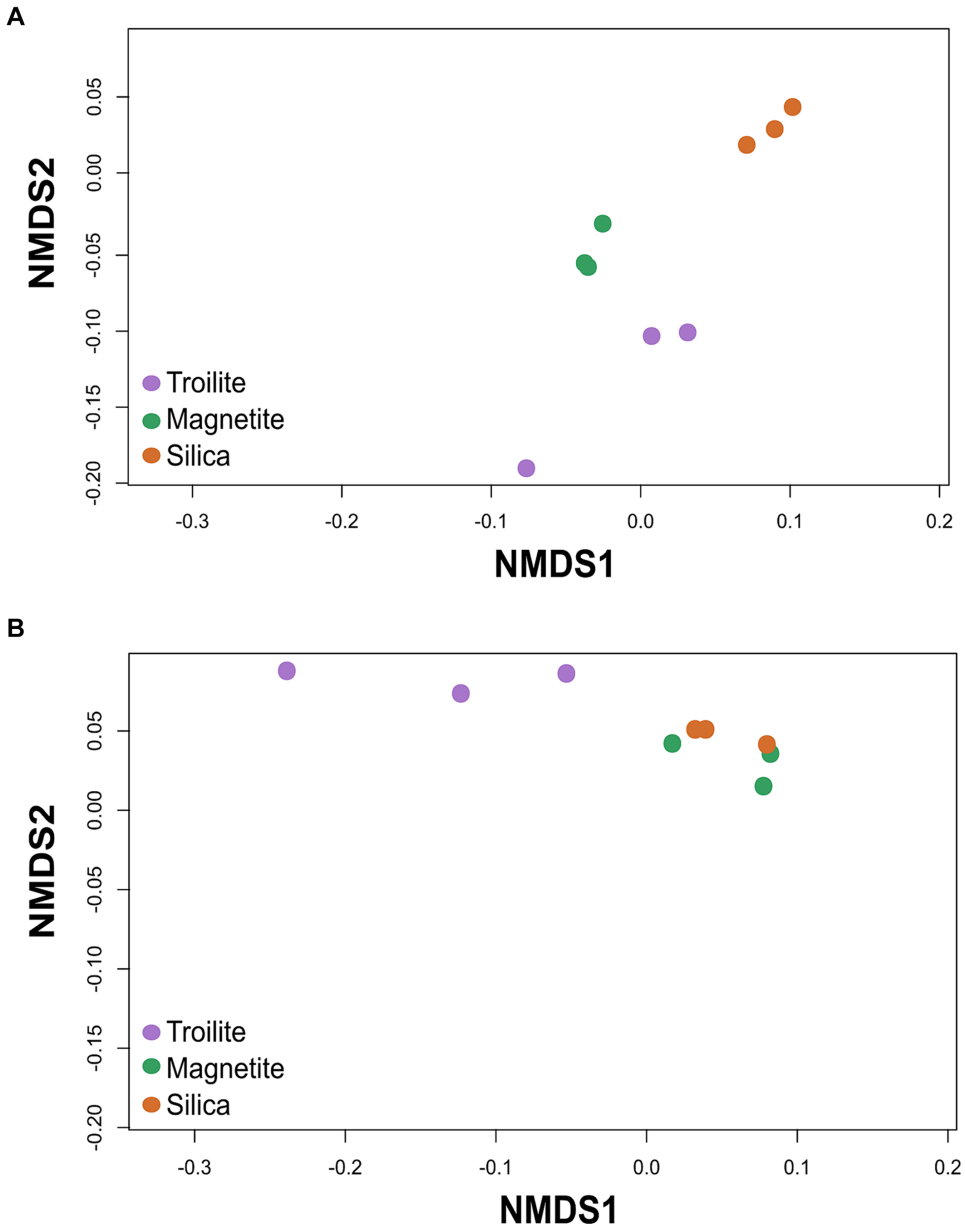
Non-metric multidimensional scaling (NMDS) of the microbial communities developed in troilite, magnetite and silica. **(A)** NMDS for total microbial communities **(B)** NMDS for active microbial communities. The analysis was based on the Weighted UniFrac distance matrices.

The NMDS analysis of microbial communities across the three different deposits is presented in Figure 4. The ordination analysis revealed differences in biofilms’ total microbial community structure formed on troilite, magnetite, and silica deposits (Figure 4A). Noticeably, greater differences were observed between biofilms formed on silica and troilite. In this analysis, samples ordinated closer to each other are likely to have more similar community structures than those ordinated farther apart. Consistent with these findings, the PERMANOVA indicated significant differences (*p* < 0.05, Supplementary Table S2) in the beta diversity of total microbial communities growing in silica versus the one developed in troilite. Moreover, despite the distance between microbial communities growing in magnetite versus silica and between magnetite and troilite, there were no significant differences in their beta diversity (*p* > 0.05, Supplementary Table S2).

The NMDS results for the active microbial communities showed an ordination where the microbial community developed in silica was clustered with the magnetite microbial community, while the microbial community developed in troilite was further apart. Statistical analysis demonstrated significant differences in the microbial community structure of biofilms developed on troilite versus the ones developed in silica and magnetite (*p* < 0.05, Supplementary Table S2). As in the total community, no significant differences (*p* > 0.05, Supplementary Table S2) were seen in the beta diversity of active microbial communities growing in magnetite versus silica. These results indicate that microbial populations formed in the silica and magnetite minerals were similar to each other and very different from those formed in troilite.

#### 3.2.3 Microbial viability

**Figure 5:**
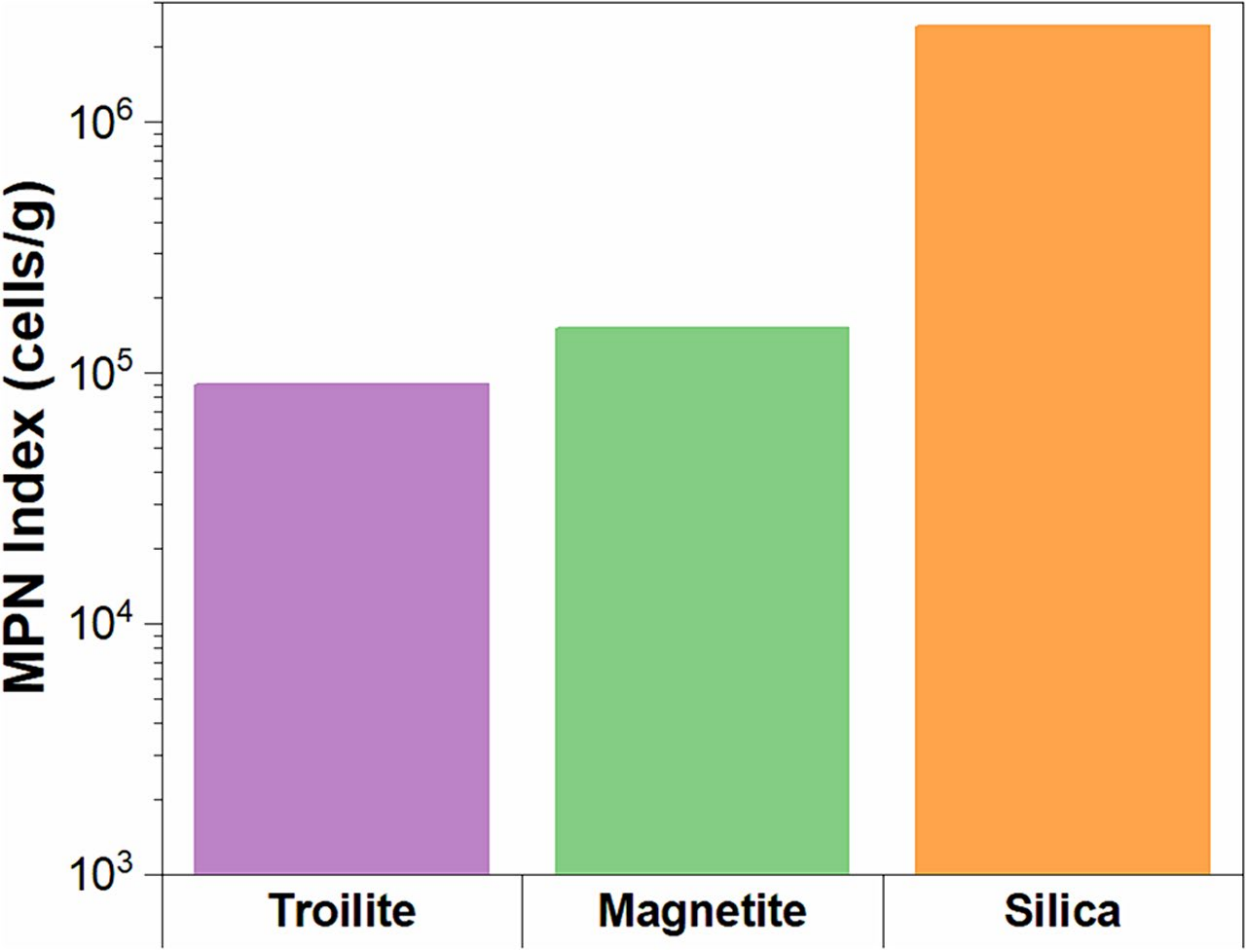
Microbial cell concentrations for biofilms formed on the three different deposits determined by the MPN method.

The microbial cell concentrations of the biofilms developed in the three different deposits are presented in Figure 5. The results show that the deposit type had a clear impact on the viability of sessile microorganisms after the two-week immersion period. Cell viability was the highest in the silica deposit (2.40 × 10^6^ cell/g), followed by magnetite (1.05 × 10^5^ cell/g), and finally, troilite led to the lowest cell viability with 9.0 × 10^4^ cell/g.

#### 3.2.4 Microbial activity levels

**Figure 6:**
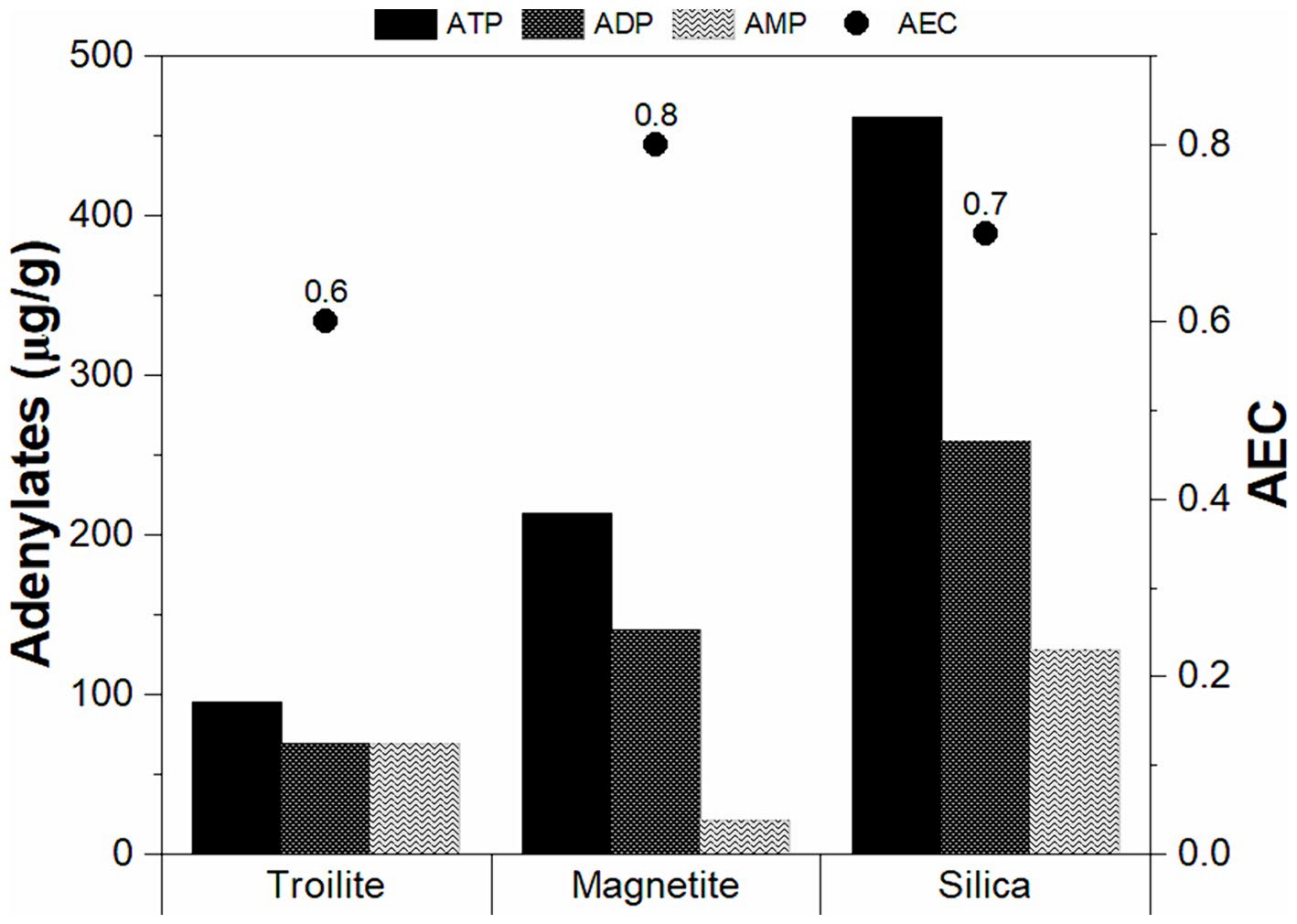
Average adenosine triphosphate percentages and adenylate energy charges of the sessile communities grown in troilite, magnetite, and silica.

The intracellular concentrations of ATP, ADP, and AMP, along with the corresponding energy charge (AEC) measurements in the biofilm developed across different deposits, are presented in Figure 6. Notably, the biofilms cultivated in the presence of troilite exhibited the lowest concentrations of ATP, ADP, and AMP, showing 94.8, 68.9, and 69.4 μg/g, respectively. In contrast, biofilms in the reactor with magnetite showed moderately higher concentrations (213.5 μg/g ATP, 140.69 μg/g ADP, and 20.69 μg/g AMP), while those in the silica displayed the highest concentrations with 461.9 μg/g ATP, 258.8 μg/g ADP, and 127.6 μg/g AMP. Statistical analysis underscored the significance of the observed differences in the relative proportions of these nucleotides across the biofilms as a result of the deposit present in the reactor, with *p*-values < 0.05 (Supplementary Tables S3–S5).

Furthermore, our findings in the adenylate concentration align with microbial cell concentration measurements (Figure 5). The biofilms in silica, with the highest concentrations of ATP and AMP, correspondingly exhibited the highest cell concentration. This correlation suggests that the elevated intracellular stored energy in the biofilms in silica may be attributed to a larger number of cells in an active state than the ones in magnetite and troilite.

The AEC values aimed to assess stress levels and the physiological state of each biofilm are also shown in Figure 6. AEC values exceeding 0.8 generally indicate metabolically active microbial populations, whereas AEC values ranging between 0.5 and 0.8 indicate stressed but viable populations (i.e., in a stationary growth phase) (Wiebe and Bancroft, 1975). In our study, the biofilms developed in magnetite displayed the highest AEC value at 0.8, followed by the ones in silica (0.7) and troilite (0.6). Notably, all AEC values fell within the range of 0.5–0.8, suggesting that, regardless of the deposit type, all sessile cells were viable but not actively growing at the conclusion of the immersion test, possibly in a stationary growth phase.

#### 3.2.5 Biofilm characterization

**Figure 7:**
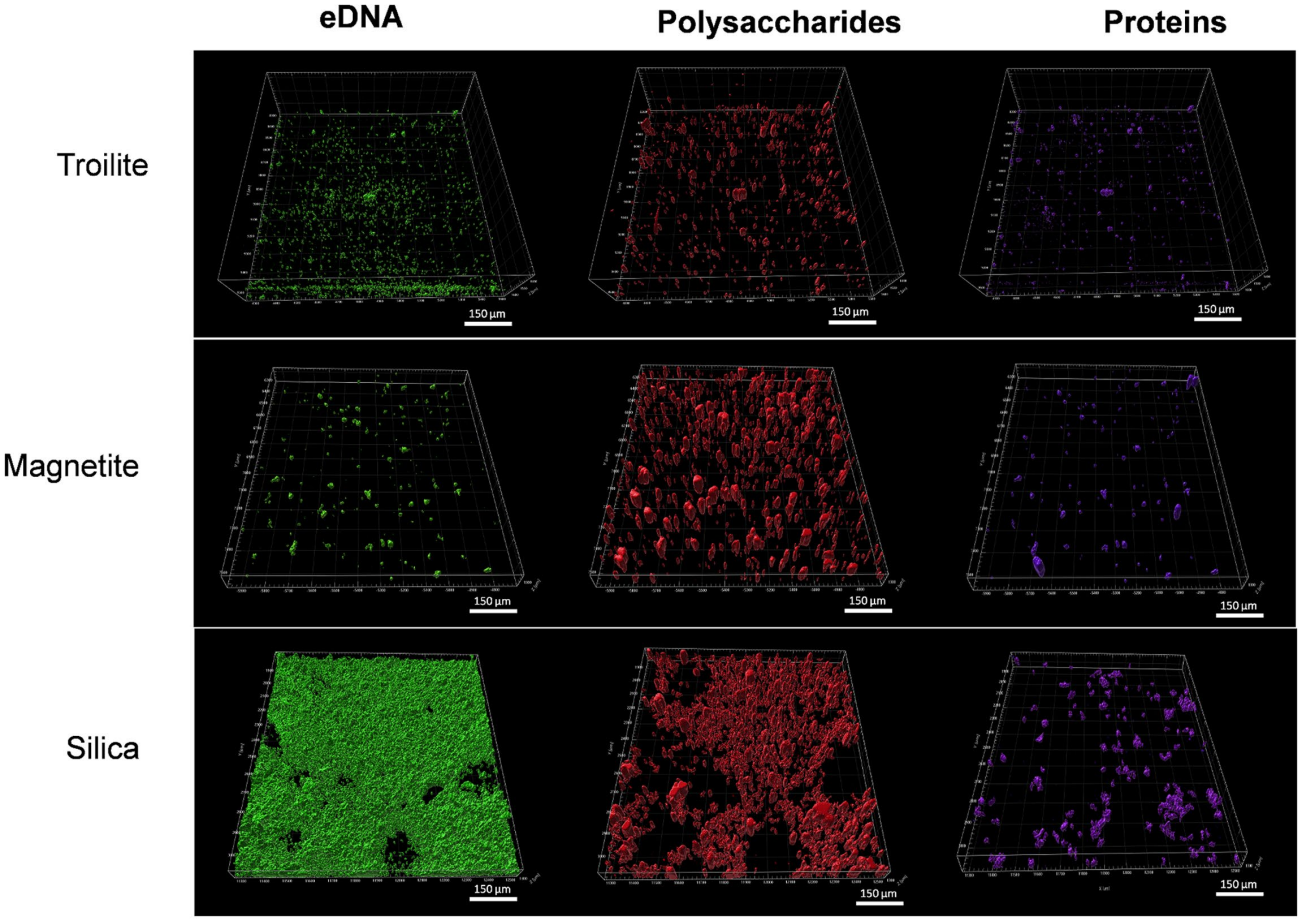
CLSM images of biofilms formed on silica, magnetite, and troilite minerals. eDNA: green stain; polysaccharides: red stain; proteins: violet stain.

Figure 7 displays CLSM images of the biofilm formed in troilite, silica, and magnetite minerals. Differentiation of biofilm components eDNA, proteins, and polysaccharides was achieved using specific stains for each component. No or negligible autofluorescence was detected in any of the minerals in the abiotic controls, as assessed by CLSM (Supplementary Figure S4), indicating no or insignificant impact of mineral autofluorescence in our samples’ differentiation of biofilm components. CLSM images revealed structural differences in biofilms influenced by the type of deposit in the reactor.

Overall, denser biofilms were observed on silica, whereas the least dense biofilms were observed on troilite. Remarkably, silica-grown biofilms were primarily composed of eDNA (as indicated by green fluorescence) and polysaccharides (as indicated by red fluorescence), with proteins (violet fluorescence) accounting for a lower proportion. In contrast, biofilms on magnetite were primarily composed of polysaccharides, with eDNA and proteins in equally low proportions. Finally, biofilms on troilite exhibited a dominant composition of polysaccharides, followed by eDNA and, in a lesser proportion, proteins. Denser biofilms were observed in microbial populations exhibiting the highest metabolic activity and cell concentrations, while less dense biofilms were observed in populations with lower activity levels and cell concentrations (Figures 5, 6).

**Figure 8:**
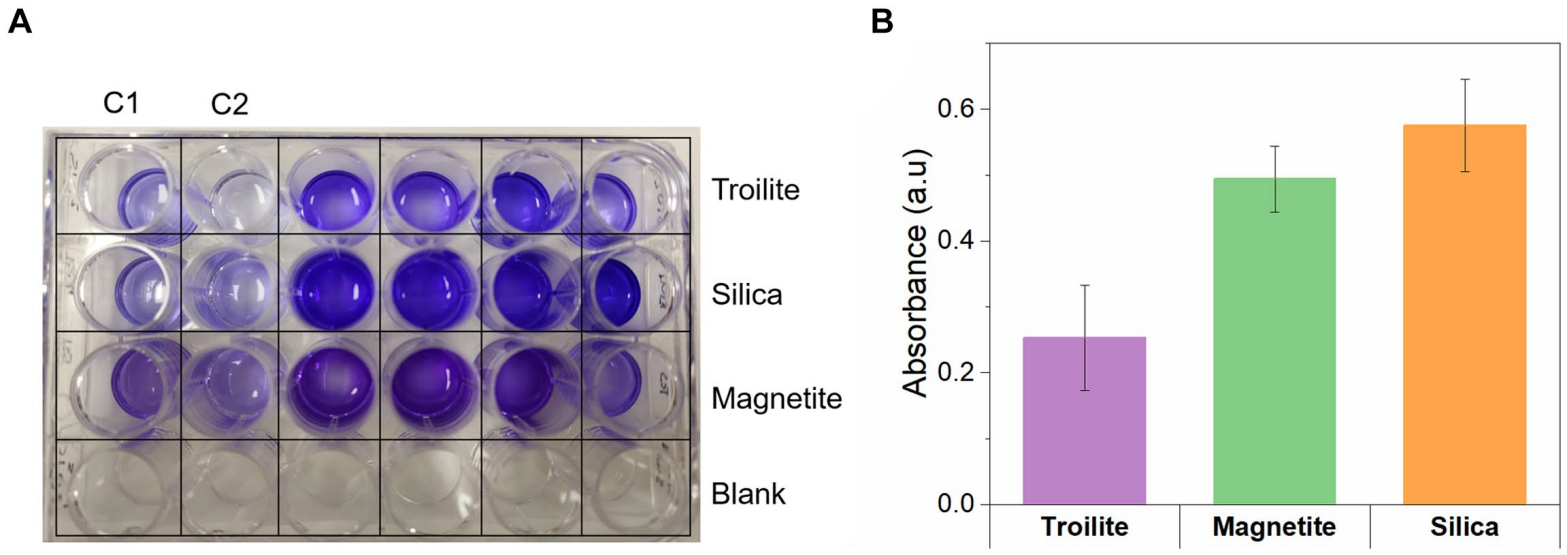
Crystal violet assay for measuring the biofilm density of biofilms formed on silica, magnetite, and troilite minerals. **(A)** Digital photo showing the color development in the 24-well microplate. **(B)** Quantitative assessment of biofilm formation determined by subtracting the average control absorbance from the average sample absorbance measured for each deposit.

The Crystal violet assay facilitates the measurement of biofilm density by binding to various biofilm components, including polysaccharides, proteins, cells, and extracellular polymeric substances (Nagaraj et al., 2017). Figure 8 provides evidence that the biofilm density of the microbial community developed in silica was the highest among the tests. Biofilms formed on silica exhibited absorbance readings 1.1 times higher than the ones measured in magnetite and 1.5 times higher than the ones measured in troilite. These results align with the conclusions drawn from the CLSM analysis (Figure 7), where denser biofilms were observed on silica, and less dense biofilms were formed on the reactor with troilite.

## 4 Discussion

This study underscored the distinct influence of three major mineral components (Fe_3_O_4_, FeS, and SiO_2_) commonly found in deposits within oil and gas pipelines on the taxonomic and physiological characteristics of an oilfield-recovered multispecies consortium. Results of this investigation demonstrated that the microbial community structure, microbial activity levels, cell viability, biofilm formation, and EPS composition are influenced by either the presence of troilite, magnetite, or silica in the environment, providing valuable insights into the interplay between minerals and microbial dynamics in oil and gas systems.

### 4.1 Role of the different minerals in controlling microbial community composition

Our results highlight the distinctive influence of troilite in shaping a distinct microbial community, diverging significantly from the communities formed in silica and magnetite, which exhibited significant similarities (Figure 4; Supplementary Table S2). Physicochemical attributes and mineral chemistry have been identified as intrinsic factors explaining how minerals shape bacterial populations across diverse environments and laboratory-based systems (Vieira et al., 2020; Diaz-Mateus et al., 2023; Kelly et al., 2023). For example, Kelly et al. analyzed the bacterial communities in two terrestrial volcanic minerals (basalt and rhyolite) and found substantial differences. There were 46 operational taxonomic units (OTUs) unique to the rhyolitic sample and 57 unique to the basaltic sample. Chao1 richness and Shannon diversity indexes indicated higher diversity and richness within the basaltic sample. The authors attributed this result to basaltic glass weathering to a secondary mineral (palagonite), whose cations may be more accessible for microorganism usage compared to solid rock. The presence of these cations, which influenced the types of microorganisms that thrived by selecting those with nutrient requirements, could be met within these cations.

While minerals, depending on their composition, mobility, oxidation state, and solubility, can weather different ions that can serve as a source of essential inorganic nutrients for the growth and sustenance of microorganisms (Byrne et al., 2015; Dong et al., 2022), they can also weather toxic ions, shaping microbial communities. For instance, Huang et al. (2021) studied the role of mineral type in the microbial communities of water samples from cooper mines versus polymetallic mines. Results showed that the higher SO_4_^2−^ and metal ions (Mg^2+^) concentrations in cooper mines differentiated the community structures. These ions reduced most heterotrophic microorganisms’ relative abundances while favoring aerobic chemoheterotrophic and iron oxidizing microorganisms.

Compared to magnetite and silica, troilite is a mineral with lower chemical stability under typical environmental conditions (Taylor, 1980; Kavner et al., 2001; Zachara et al., 2002; Oganov et al., 2005). Troilite valence bands, derived from iron and sulfide orbitals, are prone to proton attacks (acid dissolution). With the breaking of the bonding between the iron and sulfur, the liberation of HS^−^ (H_2_S) and Fe^2+^ to the environment occurs (Hu et al., 2020). The trends observed during the monitoring of dissolved sulfides and total iron concentrations in the reactor with troilite (Figure 1) and, a noticeable color change in the test solution from day two to day three suggest troilite dissolution. Differences between total and active microbial communities in the reactor with troilite support the hypothesis of troilite dissolution. The higher relative abundance of *Unclassified Enterobacteriaceae* and *Kluyvera* in the total community, compared against their relative abundance in the active microbial community (Figure 2), suggests their heightened activity at some point during the immersion period. Since members of the *Enterobacteriaceae* family are known to be involved in glucose fermentation (Octavia and Lan, 2014), these two genera were likely significantly active during the first 3 days of the test, when weak acids released as metabolic by-products of fermentation favored the dissolution of troilite by proton attack (Figure 1). The subsequent availability of Fe^+2^ and sulfur compounds in the test solution led to the dominance of *Dethiosulfovibrio*, a potential sulfide-oxidizing nitrate-reducer (Cytryn et al., 2005), accompanied by *Pseudomonas*, capable of doing iron oxidation using nitrate as an electron acceptor in the absence of oxygen (Jia et al., 2017a,b; Li et al., 2018), and also proven capable of coupling sulfide oxidation to nitrate reduction (Guo et al., 2020). Therefore, the dissolution of troilite is probably the key factor that shaped a microbial community distinct from the microbial communities formed on silica and magnetite.

Our results also indicated a higher alpha diversity index Chao1 in the reactor with magnetite for both total and active microbial communities than in the reactors with troilite and silica (Figure 3). Previous studies (Wang et al., 2019; Zhuang et al., 2020) have described an increase in alpha diversity in multispecies microbial communities with magnetite addition in anaerobic conditions. This phenomenon is attributed to magnetite facilitating direct interspecies electron transfer (DIET) between microbial species. DIET allows microorganisms to exchange electrons between them to obtain energy by facilitating reactions that no single microorganism can carry out (Shrestha and Rotaru, 2014). While the number of taxonomic groups was similar in the three different reactors (Figure 2), the higher richness found in the reactor with magnetite may be related to the exclusively presence of an uncultured Firmicutes bacterium with a relative abundance of <1%, in this reactor. Although orthologues of the genes responsible for extracellular electron transfer (EET) have been found in many species across the Firmicutes phylum (Light et al., 2018), and *Sulfurospirillum multivorans* reported the production of pili and flagella (Scholz-Muramatsu et al., 1995), structures that might contribute to DIET, further experimentation is required to confirm the role of magnetite in increasing the richness of multispecies microbial communities through DIET.

### 4.2 Role of the different minerals in biofilm formation and EPS composition

Our results demonstrate that the biofilm formation of the oilfield-recovered microbial consortium was influenced by the mineral deposit present in each test. The microbial population grown in silica displayed the highest formation of biofilm (Figures 7, 8), followed by the one developed in magnetite, finally, the microbial population grown in troilite exhibited the lowest biofilm formation. Notably, an association was evident between biofilm formation, cell concentration, and microbial activity levels across the three microbial communities. Biofilms developed in silica showed the highest microbial cell concentration, followed by magnetite and troilite (Figure 5). Consistently, biofilms developed in silica exhibited the highest intracellular ATP concentration, followed by the ones in magnetite, and finally, biofilms developed in troilite showed the lowest ATP concentration. This suggests that the higher number of cells in the silica likely contributes to higher biofilm formation and ATP concentrations compared to the ones grown on magnetite and troilite.

The higher viability of sessile cells grown on silica compared to the ones grown on magnetite is consistent with previous studies with single-species biofilms (Asadishad et al., 2013; Cai et al., 2013; Wu et al., 2014a). Asadishad et al. (2013) determined *E. coli* D21f2 and, *E. coli* O157:H7 inactivation rates over time after attachment to silica and iron oxide (Fe_2_O_3_) particles, finding an initial viability over 90% that decreased over time. At the end of the experiment, the iron oxide-bound sessile cells exhibited up to one-time higher inactivation rates compared to silica-bound cells. The authors attributed this difference to a greater number of covalent bonds, particularly, C-metal or O-metal bonds in the iron oxide over the silica, which enhanced bacterial attachment. Moreover, the tight binding of microbial cells with iron oxides by strong electrostatic attraction forces have been proposed as capable of piercing into the cell membranes causing cell death, and impeding nutrient uptake and metabolite efflux (Cai et al., 2013). Simultaneously, the lowest cell viability (9.0 × 10^4^ cell/g) measured on sessile cells grown on troilite compared to the ones grown on magnetite (1.05 × 10^5^ cell/g), and silica (2.40 × 10^6^ cell/g) is likely associated with the dissolution of troilite, which increased the concentration of total iron and sulfides in the test solution (Figure 1). Sulfide ions in the test solution may have reacted with various metal ions present in the culture media, forming metal sulfides that encapsulate the microorganisms, thereby preventing their physical contact with electron donors, acceptors, and nutrients (Utgikar et al., 2002).

Simultaneously, it is important to note that microbial interactions with solid surfaces are highly influenced by surface properties such as specific surface area, particle size, hydrophobicity, and surface charge (Hong et al., 2012; Cai et al., 2013; Wu et al., 2014b). Previous studies focussed on biofilm formation have evidenced that higher specific surface area (smaller particle size) of solid surfaces favors biofilm formation because it represents a higher total area for microbial colonization (Borcherding et al., 2014; Parker C. W. et al., 2018; Parker S. P. et al., 2018; Wu et al., 2022). For instance, Ma et al. (2017) demonstrated that *Bacillus subtilis* produced the highest biofilm mass, in terms of biovolume and thickness on goethite powders, which had a specific surface area of 79.9 ± 1.1 m^2^ g^−1^, when compared to the biofilm mass developed in montmorillonite and kaolinite, which reported surfaces areas were 55.3 ± 8.5 and 21.2 ± 0.9 m^2^ g^−1^, respectively. Moreover, the lowest biofilm mass was evidenced in kaolinite. In our study, silica exhibited the highest specific surface area among the tested minerals (Table 1), suggesting there is a potential impact of its surface area on our biofilm formation results. Nevertheless, our results do not prove a causative effect, and further studies are needed to better understand the link between the nature of minerals with the higher cell viability and biofilm formation.

Simultaneously, the EPS composition was also influenced by the type of deposit in the reactor. Biofilms on silica were predominantly composed of extracellular DNA (eDNA), while the ones on magnetite and troilite were predominantly composed of polysaccharides. These results align with previous studies that reported changes in the biofilm components as a consequence of environmental conditions such as solid surface properties (Forson et al., 2020), nutrient availability (Myszka and Czaczyk, 2009), presence of toxic compounds (Mu et al., 2012), temperature and pressure (Wang et al., 2018), and the microbial strains making up the biofilm (Guillonneau et al., 2018). Nevertheless, statistical analyses of this study indicated similarity in the total microbial communities of troilite and magnetite and the active microbial communities between silica and magnetite (Supplementary Table S2). Therefore, the variations in EPS composition are unlikely to be related to specific microbial species. The authors hypothesize that the higher eDNA content in silica-grown biofilms is directly associated with the higher number of cells per gram of mineral found in silica compared to magnetite and troilite. This hypothesis is based on previous research, which has established that the main sources of biofilm eDNA are (1) active release by microorganisms and (2) microbial lysis (Ibáñez de Aldecoa et al., 2017; Kim et al., 2018).

### 4.3 Microbial taxonomic and physiological changes in response to mineral deposits: implications for UDMC

The taxonomic profile of microbial communities in oil and gas systems is recognized as a critical factor influencing the severity of MIC (Bonifay et al., 2017). This influence does not stem from the ability to attribute a specific corrosion rate or risk to a single microbial species but rather from the metabolic capabilities inherent to each microbial species, which drive the different corrosion mechanisms. In our study, we observed the emergence of three distinct active microbial communities by the conclusion of the immersion period (Figure 2). *Dethiosulfovibrio*, a microbial genus known for its ability to utilize elemental sulfur and thiosulfate as electron acceptors, predominated within the active microbial community in the reactor with troilite. Conversely, *Sulfurospirillum*, a highly metabolically flexible microbial genus capable of reducing sulfur, thiosulfate, and nitrate, emerged as the dominant genus within the reactors with magnetite and silica. Although species from both genera have been linked to corrosion (Magot et al., 1997; Bermont-Bouis et al., 2007; Lanneluc et al., 2015), the potential corrosion damage that the microbial species recovered in our study can cause in the presence of deposits cannot be predicted. MIC mechanisms are significantly influenced by factors such as nutrient availability, temperature, atmosphere, and interactions among members of the microbial community. Consequently, under deposit corrosion testing with each microbial community is imperative to ascertain their corrosive potential.

On the contrary, the varying levels of microbial activity observed among the three distinct microbial communities in our study could potentially be linked to specific corrosion risks. Previous studies have linked elevated microbial metabolic activity to increased localized corrosion rates (Salgar-Chaparro et al., 2020a,b). This correlation is logically inferred, particularly in the discussion of chemical MIC, wherein heightened metabolic rates directly correlate with greater concentrations of corrosive metabolites released at the metal-microbial interface. When combined with the presence of deposits acting as mass transfer barriers, this phenomenon creates an optimal environment for the formation of aggressive microenvironments, ultimately leading to elevated corrosion rates (Yang et al., 2022). Consequently, based on the findings of our study, it is plausible that the microbial community thriving in the silica deposit may induce higher corrosion rates in the presence of deposits due to its highest recorded metabolic activity (Figure 6). Additionally, the heightened biofilm formation by the microbial community thriving in the silica deposit may result in the creation of a denser and less permeable diffusion barrier. This not only facilitates the concentration of metabolically corrosive species at the metal-deposit interface but also poses challenges to the efficacy of biocides, potentially exacerbating UDMC rates. Biofilms formed within deposits exhibit high resistance to chemical treatments aimed at mitigating MIC, primarily due to the limited penetrating power of most biocides (Licina, 2004).

Finally, the higher production of polysaccharides and eDNA by the microbial community developed in the silica compared to the magnetite and troilite communities could also be associated with potential higher corrosion damage. Polysaccharides and eDNA, negatively charged components, are expected to bind cationic compounds (Beech et al., 2005). During corrosion, Fe^+2^ released from the corroded metal might accumulate in higher concentrations within the biofilms on silica at the metal-deposit interphase, thereby accelerating the cathodic corrosion reaction at a faster rate than the biofilms developed in troilite and magnetite.

## 5 Conclusion

This investigation underscores the influence of the mineralogy of deposits commonly found in oil and gas systems on the microbial community structure of sessile cells. Minerals capable of leaching different ions can shape microbial communities by selecting microorganisms capable of adapting to the new environmental conditions generated by these minerals. In our study, troilite induced the growth of a statistically distinct microbial community compared to silica and magnetite. This divergence was attributed to the chemical instability of troilite under our tested conditions, resulting in the liberation of ferrous iron and sulfides into the test solution. Microorganisms with the potential to metabolize these ions thrived, while those inhibited by these ions remained dormant. Our experimental results revealed differences in the number of cells, energy levels, and biofilm formation between the three conditions. The reactor with silica exhibited a higher number of sessile cells with elevated intracellular energy levels, leading to the highest biofilm formation among the microbial communities over the reactors with magnetite and troilite. Finally, the CLSM results revealed changes in biofilm composition as a result of the minerals present in the reactors. The predominant compound in biofilms developed on silica was eDNA, while polysaccharides dominated the composition of magnetite and troilite-grown biofilms. These insights contribute to a deeper understanding of the intricate relationships between deposits in oil and gas systems and native microbial communities. The impact of substratum composition on the physiological status and biofilm characteristics of native microbial communities is illuminated, offering valuable perspectives for future research and practical applications in oil and gas infrastructure management.

## Data availability statement

The datasets presented in this study can be found in online repositories. The names of the repository/repositories and accession number(s) can be found at: https://www.ncbi.nlm.nih.gov/, PRJNA1089333.

## Author contributions

MD-M: Writing – original draft, Methodology, Investigation, Formal analysis, Conceptualization. SS-C: Writing – review & editing, Supervision, Project administration, Methodology, Funding acquisition, Conceptualization. JT: Writing – review & editing, Software, Methodology. HF: Writing – review & editing, Supervision, Funding acquisition.
