# Supplementary material for: Exploring the influence of deposit mineral composition on biofilm communities in oil and gas systems
Source: Front Microbiol. 2024 Jul 30;15:1438806. doi: 10.3389/fmicb.2024.1438806 (PMC11319257; doi:10.3389/fmicb.2024.1438806)
Supplement: Supplementary file 1 [file Data_Sheet_1.docx]

Supplementary Material

# Supplementary Data

**Table S1**. *P-*value calculated by the Kruskal-Wallis test and Dunn's post hoc pairwise comparison of the alpha diversity metrics of biofilms in the three different deposits tested.

| Comparison | | *p*-value | |
| --- | --- | --- | --- |
| Group 1 | Group 2 | Chao1 index | Shannon Index |
| T_DNA | M_DNA | 0.9048 | 0.0017 |
| M_DNA | S_DNA | 0.1413 | 1.185E-09 |
| S_DNA | T_DNA | 0.2787 | 1.557E-07 |
| T_RNA | M_RNA | 1.037E-08 | 0.9702 |
| M_RNA | S_RNA | 1.378E-06 | 0.9640 |
| S_RNA | T_RNA | 0.004841 | 0.9997 |

**Table S2**.  *P*-value calculated from the statistical comparison of the beta diversity of biofilms in the three different deposits tested.

| Comparison | | *p*-value |
| --- | --- | --- |
| Group 1 | Group 2 | PERMANOVA |
| T_DNA | M_DNA | 0.080 |
| M_DNA | S_DNA | 0.897 |
| S_DNA | T_DNA | 0.010 |
| T_RNA | M_RNA | 0.001 |
| M_RNA | S_RNA | 0.118 |
| S_RNA | T_RNA | 0.001 |

**Table S3**. One-way ANOVA test and Tukey’s post hoc test for multiple comparisons of ATP concentration among the microbial communities.

|  | Troilite | Magnetite | Silica |
| --- | --- | --- | --- |
| Troilite |  | 0.0004721* | 5.153E-05* |
| Magnetite | 11.43 |  | 0.01952* |
| Silica | 16.89 | 5.463 |  |

Tukey’s Q is below the diagonal, and p values are above the diagonal. Significant comparisons: p ≤ 0.05 = *

**Table S4**. One-way ANOVA test and Tukey’s post hoc test for multiple comparisons of ADP concentration among the microbial communities.

|  | Troilite | Magnetite | Silica |
| --- | --- | --- | --- |
| Troilite |  | 0.005423* | 1.266E-05* |
| Magnetite | 5.268 |  | 0.01407* |
| Silica | 9.862 | 4.594 |  |

Tukey’s Q is below the diagonal, and p values are above the diagonal. Significant comparisons: p ≤ 0.05 = *

**Table S5.** One-way ANOVA test and Tukey’s post hoc test for multiple comparisons of AMP concentration among the microbial communities.

|  | Troilite | Magnetite | Silica |
| --- | --- | --- | --- |
| Troilite |  | 5.913E-09* | 5.557E-05* |
| Magnetite | 17.92 |  | 2.567E-05* |
| Silica | 8.648 | 9.272 |  |

Tukey’s Q is below the diagonal, and p values are above the diagonal. Significant comparisons: p ≤ 0.05 = *


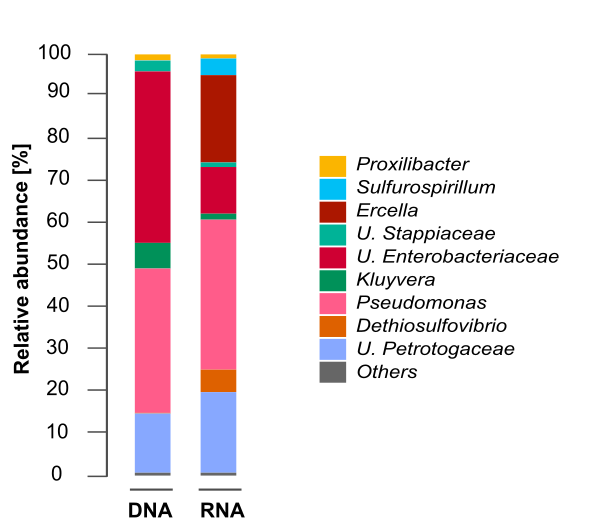


**Figure S1.** Total (DNA-based) and active (RNA-based) microbial community composition of initial. Mean relative abundances of microbial populations classified at at the genus level from 16S rRNA sequencing (*n* = 3). Genera with relative abundances lower than 1% in all samples were grouped in the “Others” category.


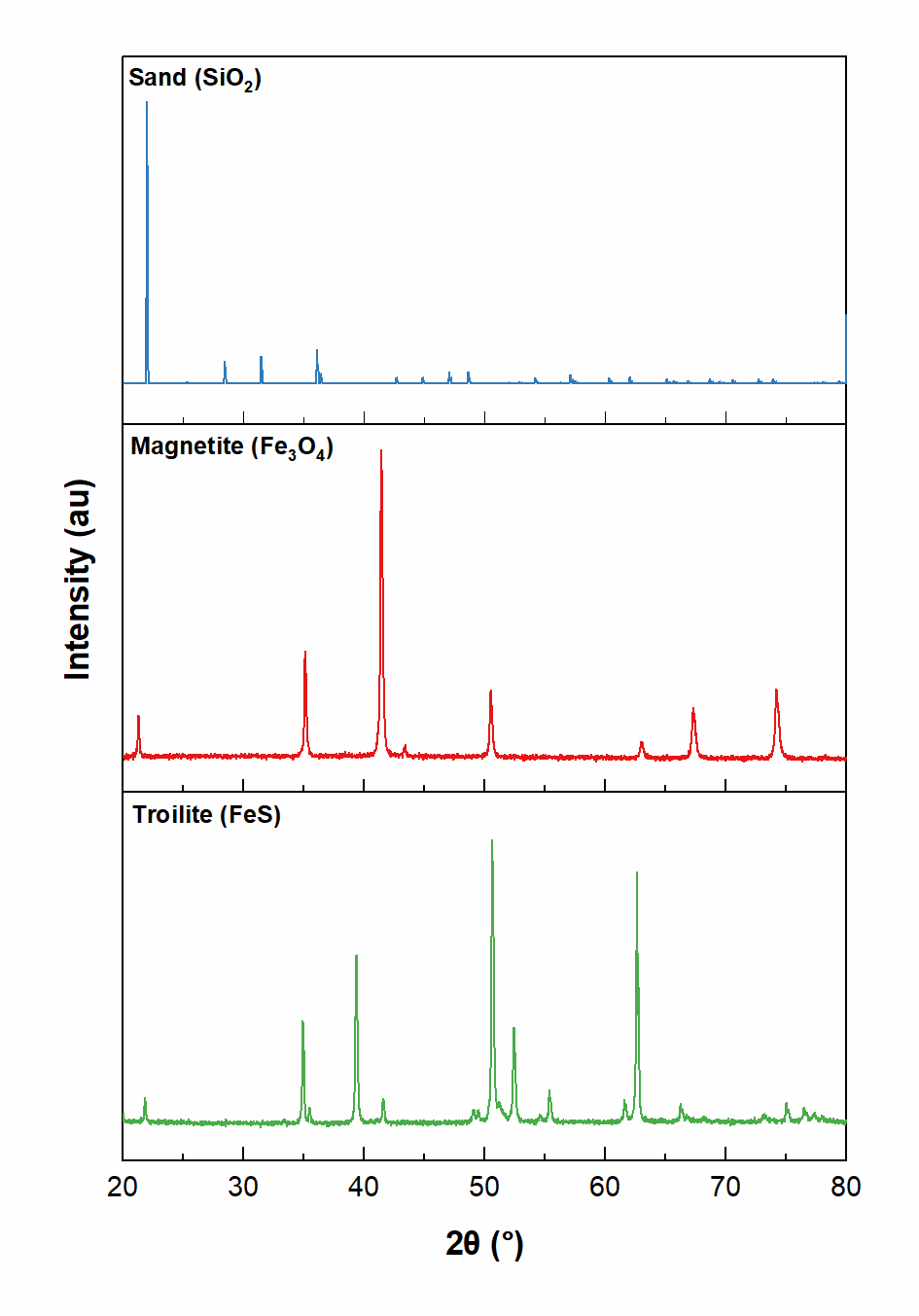


**Figure S2.** XRD spectra of the three procured minerals: Silica, magnetite, and troilite.


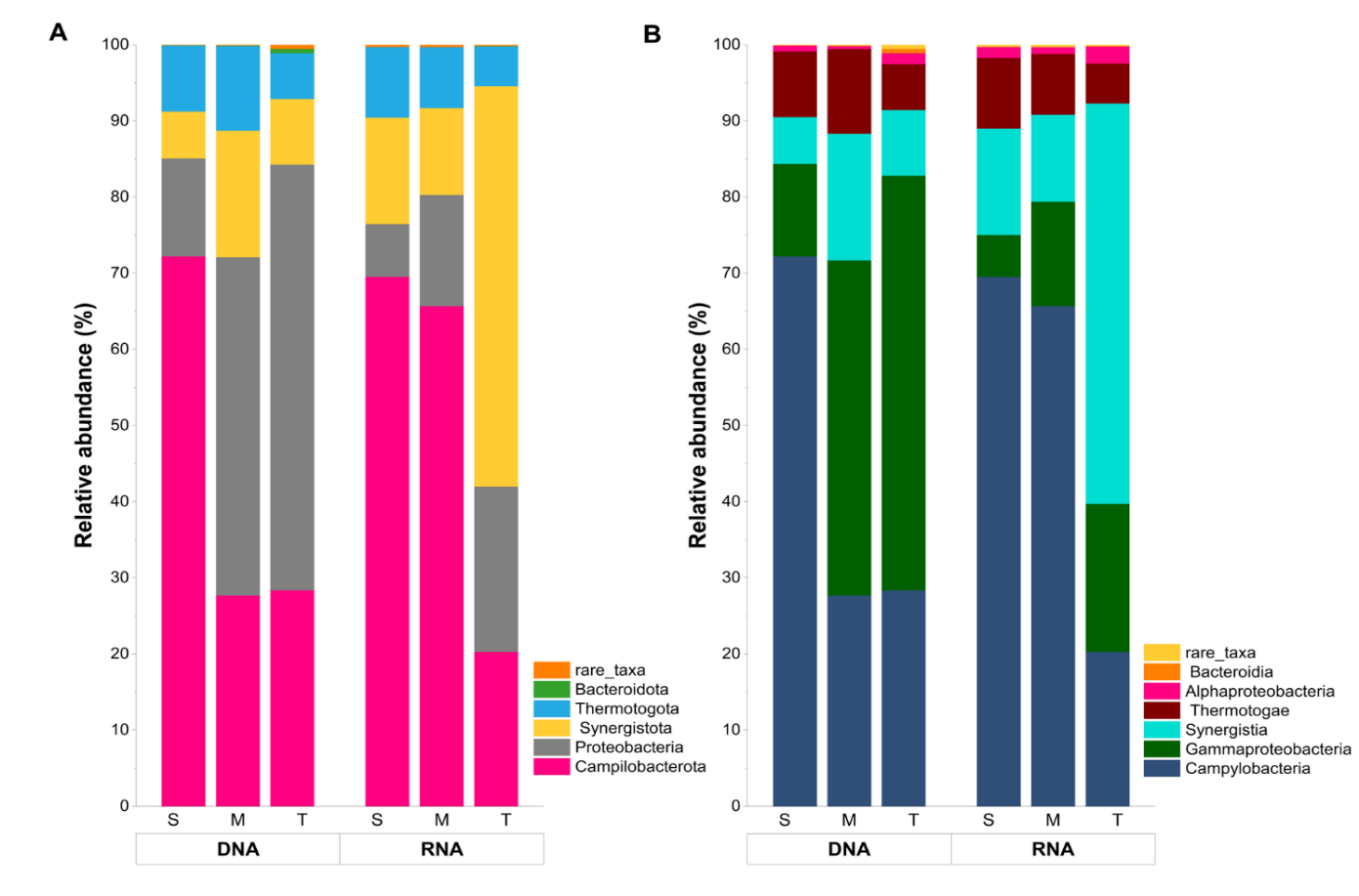


**Figure S3.** Total (DNA-based) and active (RNA-based) microbial community composition of biofilms grown in troilite (T), magnetite (M), and silica (S). Mean relative abundances of microbial populations classified at the Phylum (a) and Class level (b) from 16S rRNA sequencing (n = 3).

#
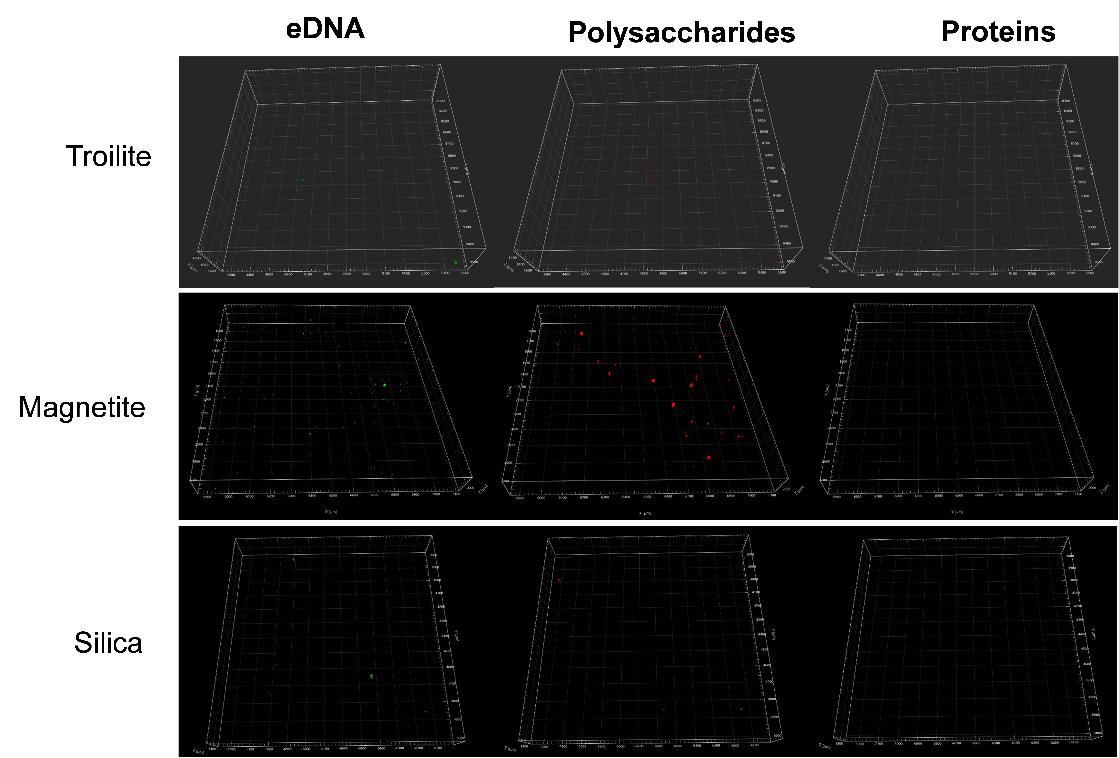


**Figure S4.** CLSM images of abiotic controls of silica, magnetite, and troilite minerals.
